# Supplementary material for: Strategies for high-temperature methyl iodide capture in azolate-based metal-organic frameworks
Source: Nat Commun. 2024 Mar 23;15:2630. doi: 10.1038/s41467-024-47035-8 (PMC10960856; doi:10.1038/s41467-024-47035-8)
Supplement: Supplementary file 1 — Supplementary Information [file 41467_2024_47035_MOESM1_ESM.pdf]

Supplementary Information

Strategies for High-temperature Methyl Iodide Capture in Azolate-based Metal-Organic Frameworks

Pan et al.

Table of Contents

Supplementary Note 1 3
Supplementary Tables 1-3..4
Supplementary Figures 1-13...7
Supplementary References 20

Supplementary Note 1

In this work, all the structural optimization was carried out under the first-principles software package DMOL3.[1, 2] The calculation method adopts the exchange-correlation functional GGA/HCTH based on density functional theory.[3] OBS method was used to correct the dispersion.[4] All calculations were performed using different orbitals for different spins, and the SCF self-consistent convergence accuracy was set to 1.0E-5 at DNP+ level. The vibration frequency of each structure was calculated during the optimization process. The free energy at different temperatures is corrected by the following formula:

$$E_T = E(0K) + G(T) \qquad (1)$$

Where, E(0K) represents the calculated single point energy of the structure at 0K, and G(T) represents the correction for free energy of the material at temperature T.

The binding energy in the reaction uses the following formula:

$$\Delta \mathrm{G} = E_T(product) - E_T(reactant) \qquad (2)$$

Supplementary Tables 1-3

Supplementary Table 1 The number of presumed adsorptive sites in MFU-Zn-X (X=Cl^-, OH^- and SCN^-) and previously reported benchmark adsorbents.

Adsorbents	Number of adsorption sites (mmol g^{-1})
MFU-Zn-Cl	3.25
MFU-Zn-OH	3.34
MFU-Zn-SCN	3.06
Ag^0-MOR	0.58
MIL-101-Cr-HMTA	8.31
COF-TAPT	11.50

Supplementary Table 2 O content, Zn content and the O/Zn ratio in MFU-Zn-OH before and after CH_3I adsorption.

	O (mmol g^{-1})	Zn (mmol g^{-1})	O/Zn
MFU-Zn-OH	18.56	4.17	4.45
CH_3I@MFU-Zn-OH	7.60	2.33	3.26

Supplementary Table 3 EXAFS Fitting results of pristine MFU-Cu(I) and two MFU-Cu(I) samples subjected to CH_3I adsorption at pressures of 0.2 bar and 0.01 bar, respectively.

	Shell	N	R (Å)	σ^2 (10^{-3} Å^2)	R factor (%)
MFU-Cu(I)	Cu-N	3.7	1.96	0.006	0.2
CH_3I@MFU-Cu(I)-0.2bar	Cu-N	2.2	2.08	0.016	0.3
	Cu-I	1.8	2.52	0.012	
CH_3I@MFU-Cu(I)-0.01bar	Cu-N	3.1	1.93	0.002	0.7
	Cu-I	0.8	2.63	0.015	

N is the coordination number; R is bonding distance; σ^2 is Debye-Waller factor; Fitting range of MFU-Cu(I): 3.00 ≤ k (/Å) ≤ 11.89 and 1.00 ≤ R (Å) ≤1.93; Fitting range of CH_3I@MFU-Cu(I)-0.2bar: 2.99≤ k (/Å) ≤ 9.80 and 1.25 ≤ R (Å) ≤ 2.80; Fitting range of CH_3I@MFU-Cu(I)-0.01bar: 2.00≤ k (/Å) ≤ 8.00 and 1.00 ≤ R (Å) ≤ 2.70.

Supplementary Figures 1-13

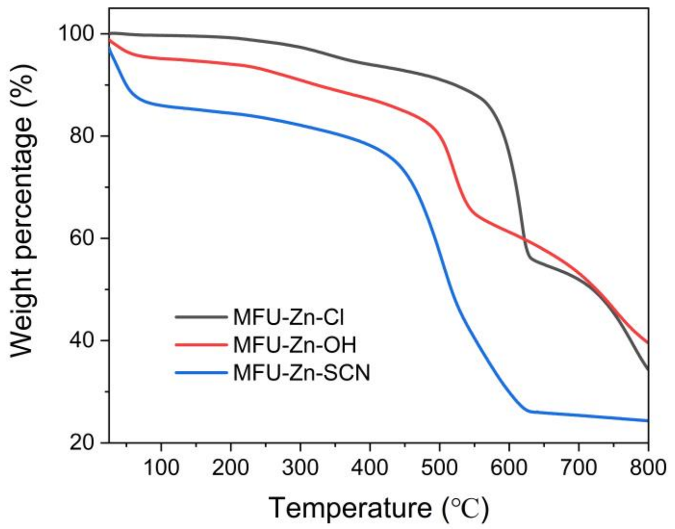

Supplementary Figure 1 Thermogravimetric analysis results of MFU-Zn-X (X=Cl, OH and SCN) recorded under a flowing N_2 gas atmosphere, with the analysis conducted over a temperature range from 25 °C to 800 °C, using a heating rate of 5 °C/min.

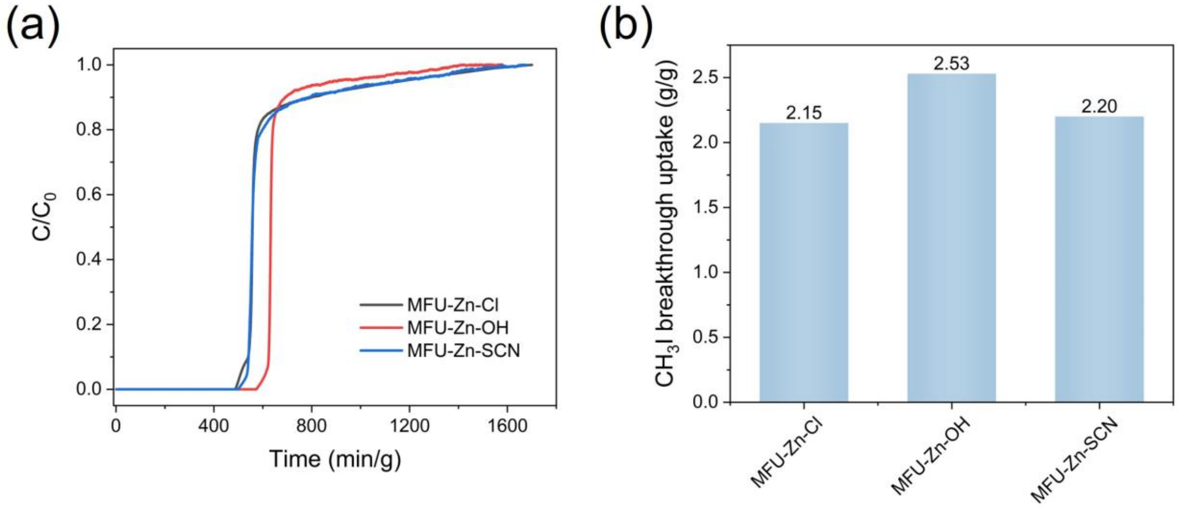

Supplementary Figure 2 Breakthrough curves of CH_3I in MFU-Zn-X (X = Cl, OH and SCN), which were obtained at 25 °C under a partial pressure of 0.2 bar (a), along with their corresponding breakthrough uptakes (b).

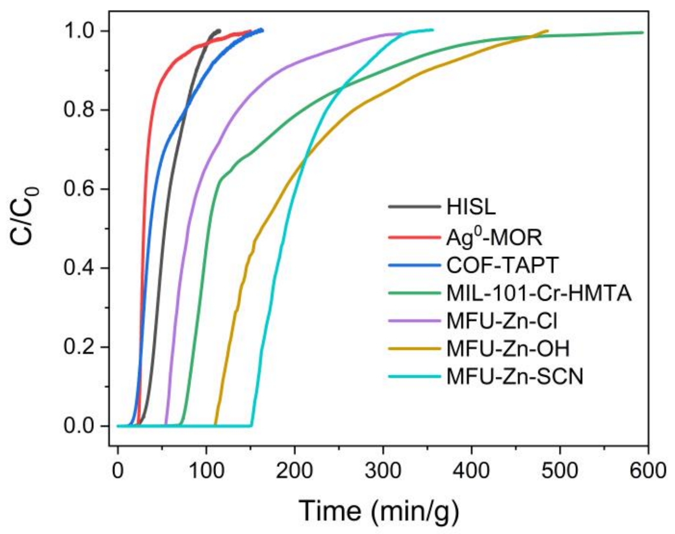

Supplementary Figure 3 Comparison of the breakthrough curves for CH_3I in MFU-Zn-X (X=Cl, OH and SCN) and benchmark adsorbents, which were obtained at 150 °C under a CH_3I partial pressure of 0.2 bar.

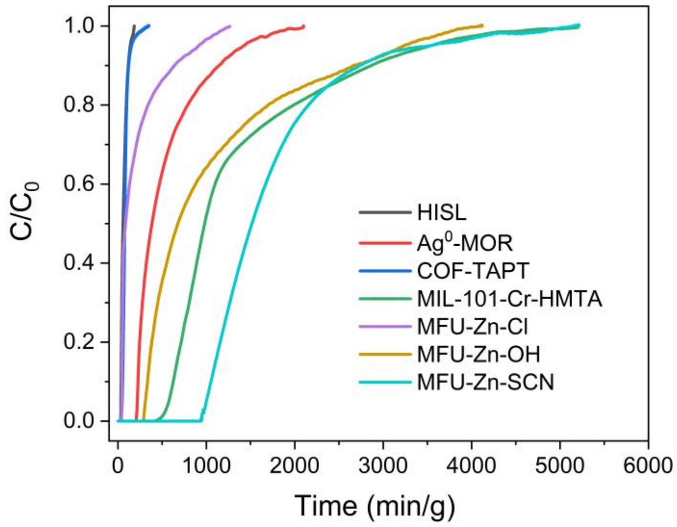

Supplementary Figure 4 Comparison of the breakthrough curves for CH_3I in MFU-Zn-X (X=Cl, OH and SCN) and benchmark adsorbents, which were obtained at 150 °C under a CH_3I partial pressure of 0.01 bar.

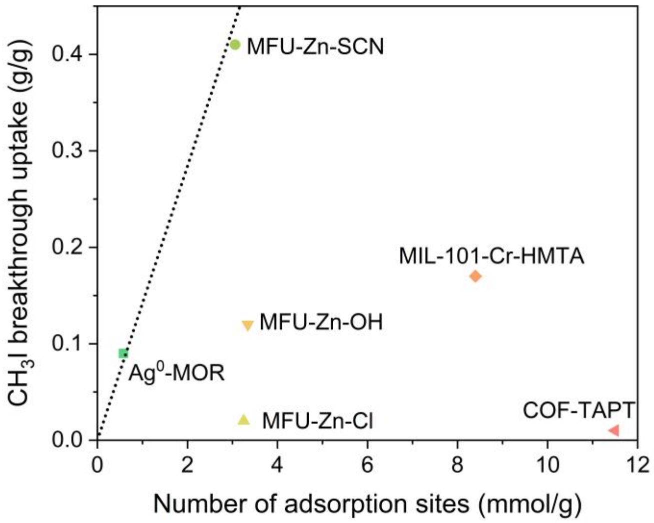

Supplementary Figure 5 CH_3I breakthrough uptake at 0.01 bar and 150 °C in relation to the number of presumed adsorptive sites across various evaluated adsorbents. The dashed straight line, plotted by the equation of q_T = (Number of adsorptive sites × 142)/1000, represents the theoretical adsorption capacity (q_T) (g/g) determined based on the number of adsorptive sites (mmol/g) and the molecular weight of CH_3I (142 g/mol), assuming a one-to-one correspondence between CH_3I and the adsorptive site.

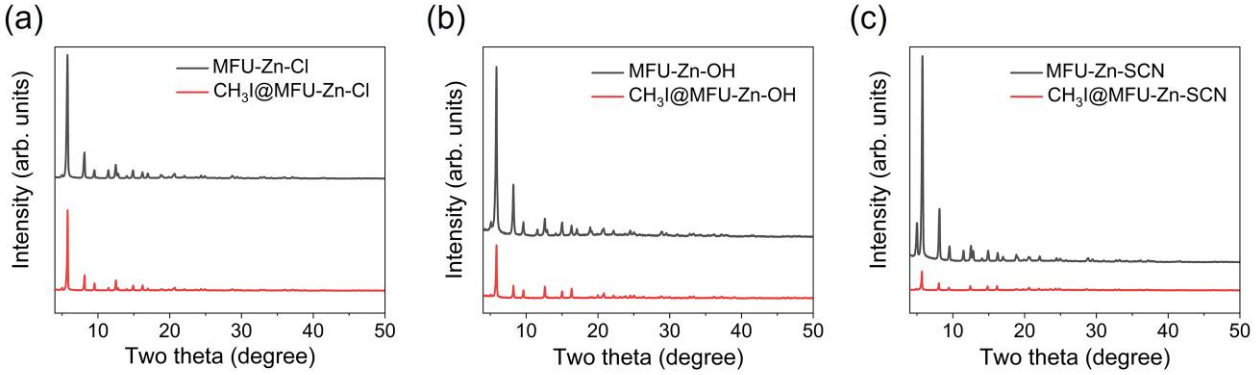

Supplementary Figure 6 PXRD patterns of MFU-Zn-X (X = Cl, OH and SCN) before and after CH_3I adsorption at 150 °C with a CH_3I partial pressure of 0.2 bar.

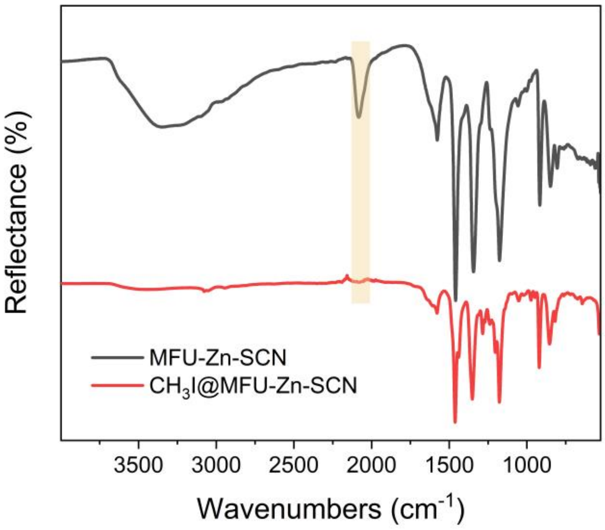

Supplementary Figure 7 FTIR spectra of pristine MFU-Zn-SCN and CH_3I-adsorbed MFU-Zn-SCN (designated as CH_3I@MFU-Zn-SCN). The highlighted band is attributed to SCN^-,[5] which disappeared after CH_3I adsorption.

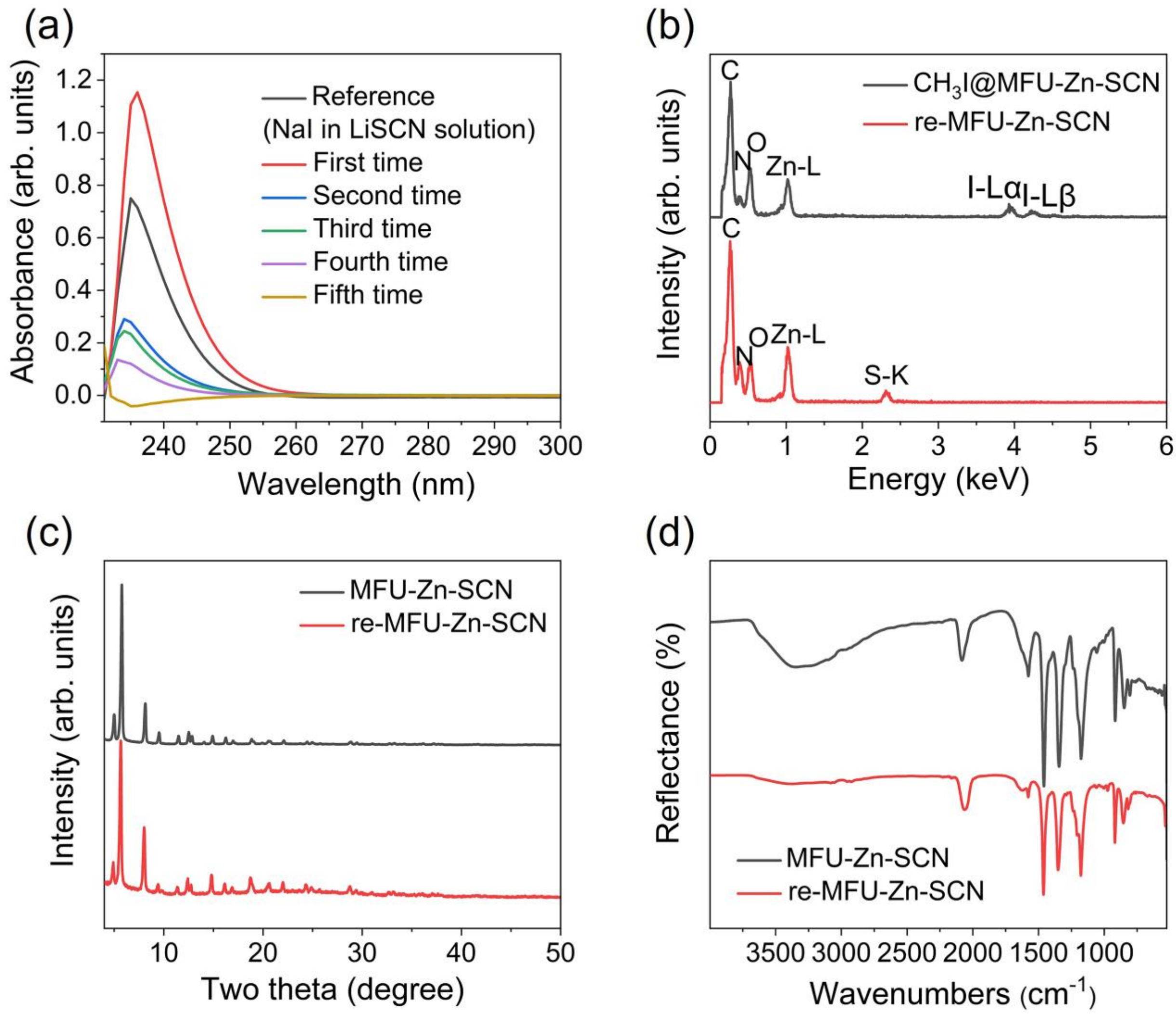

Supplementary Figure 8 (a) UV-Vis absorption spectra from five batches of a 0.2 M LiSCN aqueous solution. These batches were employed consecutively to rinse CH_3I-adsorbed MFU-Zn-SCN, aiming to regenerate the adsorbent through ion exchange. The consistent decline in absorbance across this series of spectra signifies the effective removal of iodine from the adsorbent. (b) EDX spectra of CH_3I-adsorbed MFU-Zn-SCN and the regenerated sample (denoted as re-MFU-Zn-SCN). PXRD spectra (c) and FTIR spectra (d) of pristine MFU-Zn-SCN and re-MFU-Zn-SCN.

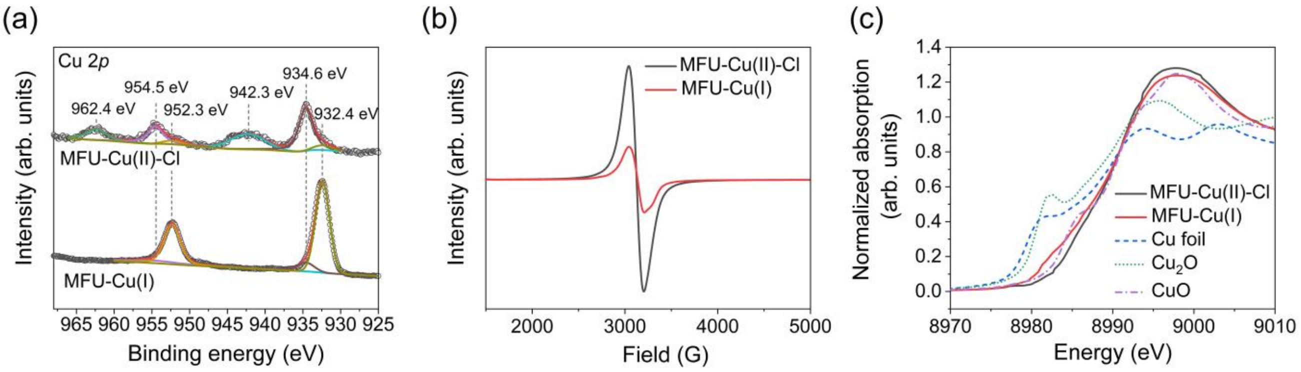

Supplementary Figure 9 (a) Cu 2*p* XPS spectra, (b) EPR spectra, and (c) Cu K-edge X-ray absorption near edge structure spectra of MFU-Cu(II)-Cl and MFU-Cu(I), along with Cu foil, Cu_2O, and CuO as reference materials. These characterization results collectively indicate the lower oxidation state in MFU-Cu(I) compared to MFU-Cu(II)-Cl.

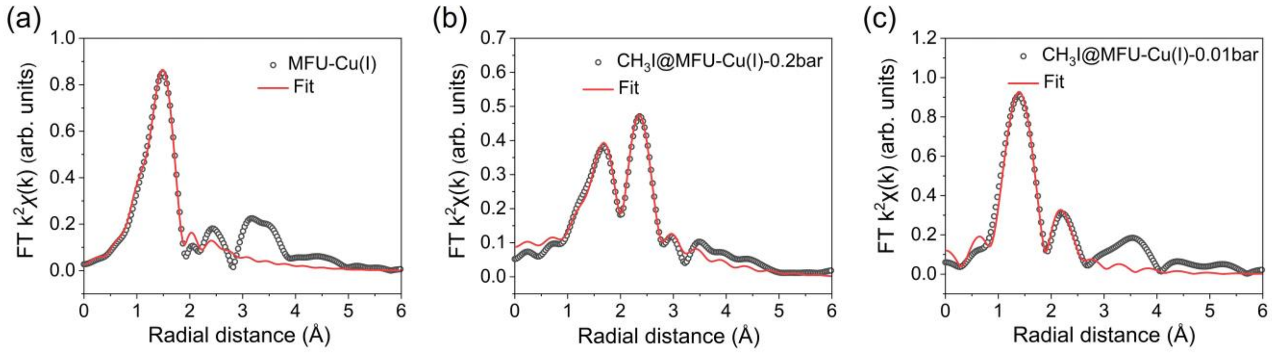

Supplementary Figure 10 (a) Cu K-edge EXAFS spectrum of MFU-Cu(I), along with the best fit, which shows one dominant peak representing Cu-N interactions. (b-c) Cu K-edge EXAFS spectra, along with the best fit, of two MFU-Cu(I) samples subjected to CH_3I adsorption at pressures of 0.2 bar (b) and 0.01 bar (c), respectively.

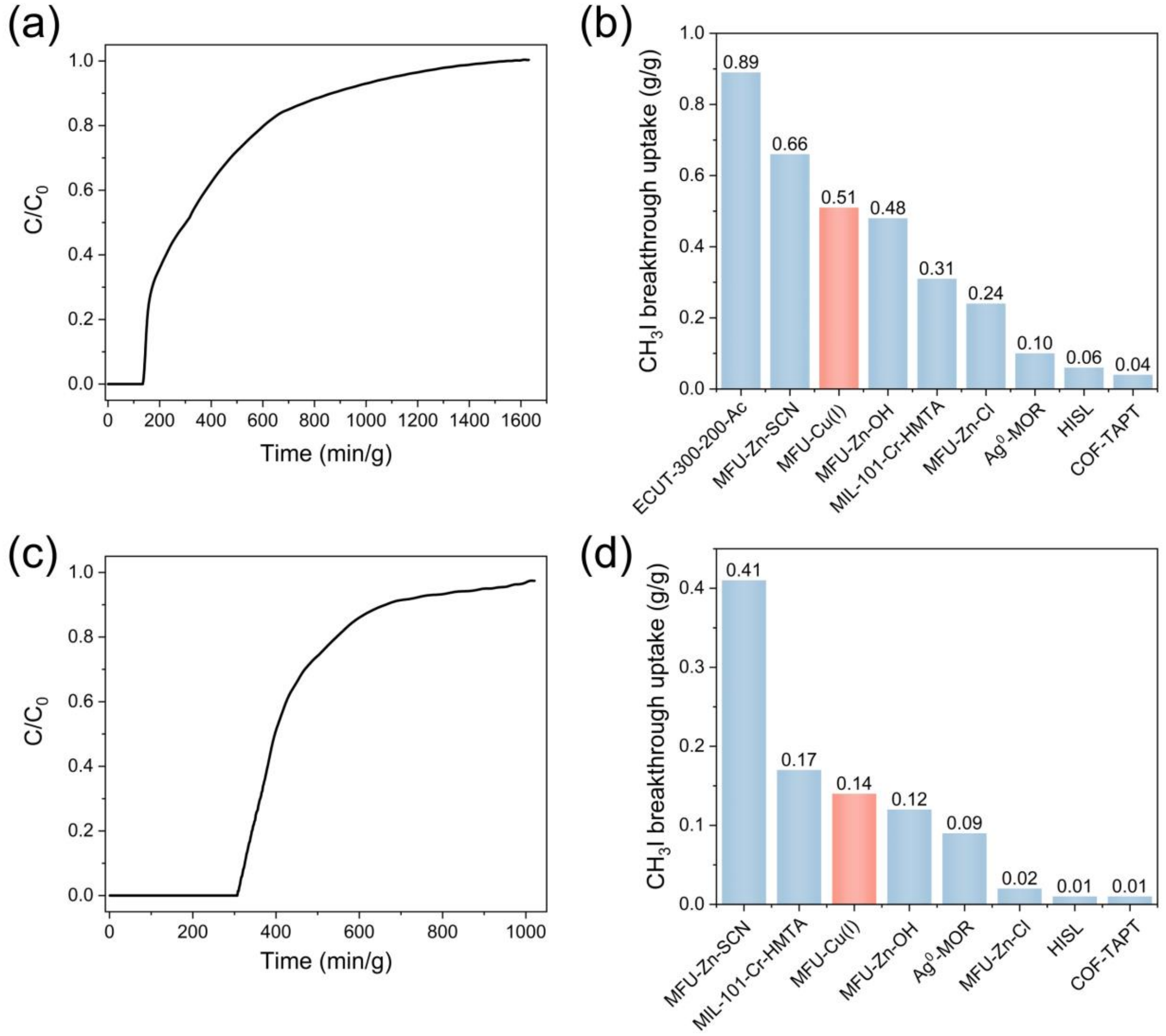

Supplementary Figure 11 CH_3I Breakthrough curves of MFU-Cu(I), obtained at 150 °C under a CH_3I partial pressure of 0.2 bar (a) and 0.01 bar (c). Comparison of CH_3I breakthrough uptakes between MFU-Cu(I) and various benchmark materials at 150 °C under a CH_3I partial pressure of 0.2 bar (b) and 0.01 bar (d).

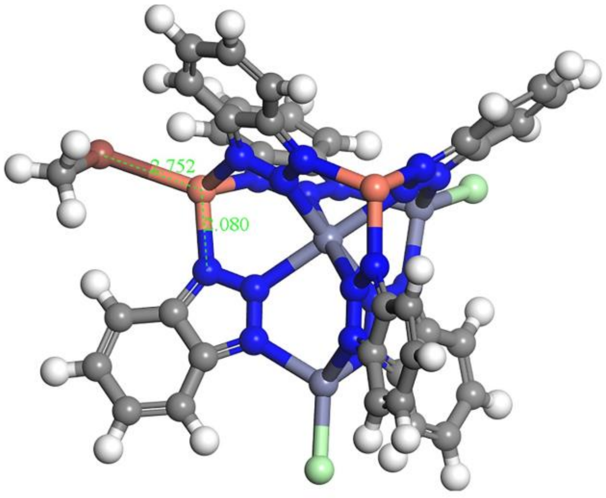

ΔG = -0.58 kcal/mol

Supplementary Figure 12 The optimized configuration of CH_3I interacting with unsaturated Cu(I) sites on MFU-Cu(I). The bond lengths of Cu-N and Cu-I are optimized to be 2.080 Å and 2.752 Å, respectively, and the binding energy is determined to be -0.58 kcal/mol.

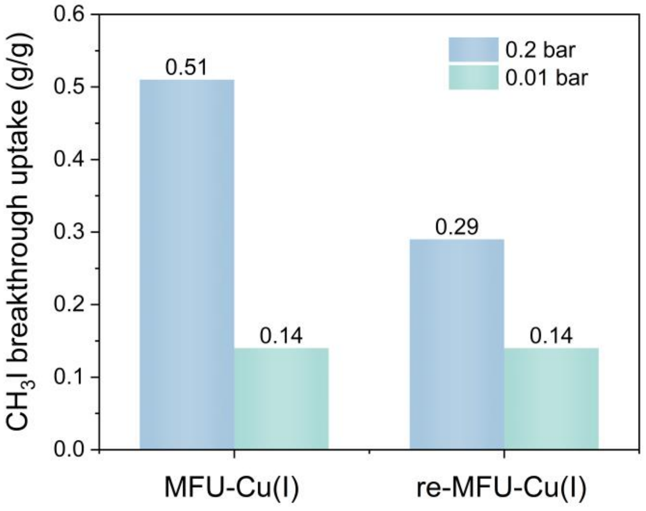

Supplementary Figure 13 CH_3I breakthrough uptakes of both fresh MFU-Cu(I) and its regenerated form, re-MFU-Cu(I), tested under high (0.2 bar) and low (0.01 bar) CH_3I partial pressures.

Supplementary References

1. Delley, B. An all-electron numerical-method for solving the local density functional for polyatomic-molecules. *J Chem Phys* **92**, 508-517 (1990).

2. Delley, B. A scattering theoretic approach to scalar relativistic corrections on bonding. *Int J Quantum Chem* **69**, 423-433 (1998).

3. Boese, A. D., Handy, N. C. A new parametrization of exchange-correlation generalized gradient approximation functionals. *J Chem Phys* **114**, 5497-5503 (2001).

4. Ortmann, F., Bechstedt, F., Schmidt, W. G. Semiempirical van der Waals correction to the density functional description of solids and molecular structures. *Phys Rev B* **73**, 205101 (2006).

5. Ganesan, K., Ratke, L. Facile preparation of monolithic kappa-carrageenan aerogels. *Soft Matter* **10**, 3218-3224 (2014).
